# The 1965 coup and *reformasi* 1998: two critical moments in Indonesia-Malaysia relations during and after the Cold War

**DOI:** 10.1186/2193-1801-3-45

**Published:** 2014-01-24

**Authors:** Ali Maksum, Reevany Bustami

**Affiliations:** Centre for Policy Research and International Studies (CenPRIS), Universiti Sains Malaysia, Penang, 11800 Malaysia

**Keywords:** The 1965 coup, *reformasi*, domestic politics, Cold War, Indonesia-Malaysia relations

## Abstract

This article discusses the significant impact of the two crucial moments in Indonesia namely, the 1965 coup and *reformasi* (reformation) in May 1998 and the impact towards the Indonesia-Malaysia relationship. History had demonstrated that both events were followed by some changes in the bilateral relationship. The 1965 coup for instance resulted the fall of Sukarno and the collapse of PKI, while *reformasi* brought the fall of Suharto and the collapse of New Order. However, it was undeniable that the demands of international situation especially during and after the Cold War were significant factor in driving of those events.

## Introduction

Some researchers have attempted to understand the extent to which the consequence of the two “special” moments, namely the 1965 coup and *reformasi* (reformation) in Indonesian politics. Edward V. Schneier argued that *reformasi* produced political transformation in Indonesia such as the end of “communism threat,” a new legislature, and the shifted of executive-legislative relations (Edward 2009). While, James H. Cassing argued that after the 1965 coup and the collapse of Sukarno, Suharto became synonymous with power and authority, mistrusted political competition and openness, and blaming these for the chaos of the Sukarno era (Cassing 2000). Both events impacted to the political scenario in Indonesian domestic politics notably in the regime changes. In the *reformasi* for instance, Suharto and his military regime collapsed after the huge mass demonstrations by university students, while the 1965 coup was an occurrence of regime transformation from Sukarno to Suharto. The coup 1965 was an abortive coup that ended with the tragic tragedy where many Indonesian people were killed, notably the members of Indonesian Communist Party (*Partai Komunis Indonesia*, PKI) the largest communist party in the world.

Both events also had a significant impact on the Indonesia-Malaysia relations due to the interplay of domestic politics in the two countries. This is because the emergence of two political factions in Indonesia and Malaysia, namely “pro” and “anti” government which indirectly influenced the relationship between the “two countries.” Thus, we offer two crucial questions regarding to what extent the factors and consequences of both events affect Indonesia-Malaysia relations. First, what both events meant for the development of Indonesia-Malaysia relations? Second, what are the factors, internally and externally, leading to the events? This article attempts to elaborate and answer these questions.

## Indonesia, Malaysia, and international politics

According to Amitav Acharya, during the Cold War era, “the vast majority of the world's conflicts occurred in the Third World and most of these conflicts were intra-state in nature (anti-regime insurrections, civil wars, tribal conflicts etc.) (Acharya 1995).” Otherwise, the conflicts and stability in the Third World’s domestic politics are also responsible to the regional stability and also contribute to the discord with neighbours (Acharya 1995:5). We argue that the roles of the domestic politics in Southeast Asia’s international relations were stimulated by the superpower engagement in the Cold War era. It was reasonable because “the superpower intervention in the Third World was subject to a set of “implicit rules of the game” which contributed to order and stability in the Third World (Acharya 1995:8).” In contrast to this, the involvement of great powers after the end of the Cold decreased, the role of domestic levels increased. This is because the international environment had changed when the communist-capitalist issue has been replaced by new issues namely globalisation.

Kenneth N. Waltz argued that in the post-Cold War era the so-called structural realism is still relevant (Waltz 2000). Specifically, Waltz argued that,” international politics is being transformed and realism is being rendered obsolete as democracy extends its sway, as interdependence tightens its grip, and as institutions smooth the way to peace (Waltz 2000:6).” The Waltz’s statement indicated that international structure affected states interest and behaviours (Mearsheimer 2007; Glaser 2003; Waltz 1979). However, it needs to be emphasized that the role of domestic politics gives an impact to states foreign relations. Because, according to Robert Jervis, “a country’s foreign policy reflects the nature of its domestic regime (Jervis 2006; Zakaria 1992; Sterling-Folker 2002).” The behaviours of Sukarno and Suharto during their administration corresponded with their foreign policies and of course with this theory.

The above concept regarding the role of domestic politics and the international system totally concurred with Gideon Rose’s concept about the role of international structure. According to Gideon Rose, international structure will effect only in a certain condition, whether in “low pressure” or “high pressure (Rose 1998).” High pressure means that states find it difficult to create foreign policy due to the high pressure from international structure in terms of international environment, alliance resolve, economic interdependence, and regionalism (Maksum 2011)^a^. By contrast, in the low pressure, states are free to conduct foreign policy (Rose 1998:152). We believe that those events have correlation with international situation and the complexity of domestic politics at the same time. The emergence of the 1965 coup and *reformasi* totally had been influenced by the dynamics of international politics. Furthermore, both events had implications for both Indonesian domestic politics and Indonesia-Malaysia bilateral relations. In the next section the 1965 Coup and *Reformasi* 1998 and its consequences upon Indonesia-Malaysia relations will be analyzed.

## The 1965 coup and the fall of Sukarno

### The background of the 1965 coup

Until recently, the speculation and the research regarding the mystery of the 1965 coup had been difficult to explain, particularly the main actors behind the coup. Some scholars like Benedict Anderson and Ruth McVey of Cornell University were involved in serious research soon after the coup (Roosa 2008). They were unable to finish the research because of limited resources and cooperation from the Indonesian military government. Fortunately, years later the Central Intelligence Agency (CIA) released documents and facts which made researchers more optimistic about the prospect of this research (Roosa 2008:27).

It was undeniable that the United States (US) played a crucial role behind the 1965 coup. Former Indonesian Ambassador to Cuba, A. M. Hanafi said that “CIA has been working long time ago since the Republic was founded, indeed (FORUM Keadilan 1999).” Thus, it was a long process and many conspirators were involved in the 1965 coup. In the Cold War era, Indonesia received special attention of the great powers (the US and its allies) because of the huge influence of Indonesian Communist Party (PKI) at the high levels of Indonesian government as well as at the grass root. Meanwhile, at the international level there emerged what the scholars call “three polar” namely, the US, Soviet Union, and China. This international situation significantly gave an impact to PKI members in the Indonesian government. After the split of Sino-Soviet relations, PKI members were prone to look at China as a better friend rather than Soviet Union though they obtained full support in securing West Irian (currently known as Papua Province).

The situation in Indonesia becomes a dilemma to communist bloc. This is because the communist’s powers (Soviet Union and China) regarded to PKI as the largest communist party in the world. However Indonesia was also under US and capitalist threat. Moreover, the split of Sino-Soviet relations affected Indonesia-Soviet relations because of Indonesian failure to repay the Soviet Union's military loans of about one billion dollars. The loan allocated by Soviet Union was assist to Indonesia’s military operation to takeover West Irian from the Dutch. To China, this situation was a good opportunity to make an approach to Indonesia by supporting *konfrontasi* (confrontation) against Malaysia in 1963 and gave approximately, USD 50 billion and allocated about USD 100 billion to establish a rival organisation of United Nations (UN) namely, NEFOS (Newly Emerging Forces) where the financial aid would be received in 1966 (Chua 2001).

The complexity of Sino-Soviet relations regarding the situation in Indonesia was utilized by US government to infiltrate into Indonesian domestic politics scenario. One of the important US operations is to stop financial assistance to Indonesia. To the contrary, the aid to Indonesian Army (TNI-AD)^b^ significantly increased. The US authority argued:

The only US aid we are now going to Indonesia consist of funds to pay for military training. We feel such training is in our interest because it helps to tie us closer to Indonesia military leaders who may well play a major role in the decision as to the future political orientation of the country.

The President interrupted to say that all US military assistance going to Indonesia is being provided because it is our national interest, not theirs. He hopes that those present would make this point clear (Harsutejo 2003).

In the meantime, the US was pessimistic towards Indonesia because they failed in their approaches to Sukarno. The US had attempted to approach Sukarno through some economic assistance. They proposed such regional forums such as Association of Southeast Asia (ASA) and the South-East Asia Friendship and Economic Treaty (SEAFET) (Liow 2005). However, all those attempts were opposed by Sukarno (Liow 2005:83–86). The US was forced to utilize his residual aid as persuasive levers which did not work due to Sukarno’s pride use in his retort “go to hell with your aid (Chua 2001:26).”

The US was worried about Indonesia because of the growing of the communists and its good prospects in the future along with its rich natural resources at the same time (Roosa 2008:16-17). Richard M. Nixon convinced that “with its 100 million people, and its 3,000-mile arc of islands containing the region's richest hoard of natural resources, Indonesia constitutes by far the greatest prize in the Southeast Asian area (Nixon 1967).” Hence, the US government had attempted to take strategic actions in handling Indonesia to prevent a takeover by communist groups. John Roosa argued that “from 1958 to 1965 the United States had trained, funded, advised, and supplied the army which could be designed as a state within a state (Roosa 2008:252).” The US government via the National Security Council (NSC) argued that “the civil power in the non-communist parties under support of the Army could turn against the communist party in the political arena (Roosa 2008:257).”

At the grassroots level, there had existed “sentiment” and tension between communist groups and Islamic groups due to the endorsement of the Land Regulations (*Undang-Undang Pokok Agraria,* UUPA) by Indonesian government which was perceived as a victory to PKI. The PKI member’s disrespect towards Islamic religion became a significant factor to the emergence of the tensions. At the highest level of Indonesian politics there emerged the tension because of the rise of the issue of *Dewan Jenderal* (General Council) which was suspected to attempt a coup against Sukarno's government. The *Dewan Jenderal* issue became serious after it was uncovered the “Gilchrist Document” at the British Embassy in Jakarta (Roosa 2008;:300 & Soebandrio 2001:18-19). Moreover, the issues of the establishment of *Angkatan ke V* (the Fifth Forces) and the armament support from China were creating a tension between PKI and the Army (TNI-AD). The PKI-TNI/AD tensions were more complicated after a broadcast was made that President Sukarno was sick and probably would die. Those situations emerged because of the provocation by the US and through some news agency such as the British Broadcasting Centre (BBC), Reuters, Radio Malaysia, Radio Australia and the Voice of America (VOA) (Roosa 2008:250–290).

In October 1st, 1965, the 1965 coup, a tragic tragedy occurred and many Indonesian people particularly those suspected as communist activists were killed. Afterwards, in the early 1966 several scholars were involved in serious research regarding the actual number of the victims of the massacre. Stanley Karnow of Washington Post said that the victims are about 500,000 in Java and Bali, while S. King of New York Times noted in May 1966 that about 300,000 people were killed (Roosa 2008:30). A Fact Finding Commission that was established by President Sukarno announced that 78,000 were killed. Indonesian former President Abdurrahman Wahid said that 500,000 PKI activists were killed by Islamic groups. However, Noam Chomsky's analysis based upon the facts released by CIA found that 250,000 people were killed. Robert Cribb also recorded in his research “*The Indonesian Killings, 1965–1966: Studies from Java and Bali*” mentioned that between 150,000 and 2 million people were killed (Cribb 1990:8-12).

### The 1965 coup and konfrontasi

The *konfrontasi* of Indonesia against Malaysia in 1963 could be considered an important factor that led to the occurrence of the 1965 coup. It was because this policy was not totally supported by all Indonesian. The *konfrontasi* is unilateral policy had created by Sukarno and supported by PKI. Many Indonesian military opposed the *konfrontasi* policy particularly those who came from TNI-AD military officers. The confused political situation was used by such individuals in Indonesian army as the anti-Sukarno groups to take an initiative to launch efforts to end the *konfrontasi*. Secretly, several Indonesian Army officers attempted to contact Malaysian authority or some Indonesian suspects (in Sukarno’s view) in Malaysia and Singapore such as General Benny Murdani, Des Alwi, Jan Walandow, and Prof. Sumitro Joyohadikusumo who were involved in PRRI/Permesta rebellion in West Sumatra.^d^ According to Des Alwi who was a key figure in the “approach” with Malaysia, the first mission failed under Syarnubi Said and Sukendro to consolidate with Ghazali Shafie of Malaysia under the initiative of General Ahmad Yani (Othman et al. 2009). Concurrently, in Indonesia occurred the 1965 coup where General Ahmad Yani as the Chief of Indonesian Army, was kidnapped and murdered together with other six generals of Indonesian military and a first lieutenant. The consolidation with Malaysia was postponed for a while because all the Indonesian elements focused on the 1965 coup. Finally, both countries reached an agreement in 1966 after Adam Malik of Indonesia and Tun Abdul Razak of Malaysia met in Bangkok, Thailand. At the same time after the ending of *konfrontasi*, both countries along with Thailand, Singapore, and the Philippines agreed to establish the Association of Southeast Asian Nations (ASEAN) in 1967.

### The 1965 coup and konfrontasi: from systemic factors to Indonesian domestic politics

Based on the above discussions regarding the coup 1965, it can be established firstly, the Cold War situation had influenced the political configuration in Indonesia. The Sukarno policy to build diplomatic relations with communist bloc rather than capitalist bloc had affected the Indonesian domestic politics which also influence the foreign relations. The PKI itself faced a dilemma because of the split of Sino-Soviet relations that indirectly influenced the Soviet Union behaviour towards Indonesia. The Soviet Union-Indonesia relations during *konfrontasi* cooled down (Chua 2001:26). Secondly, the Sukarno’s *konfrontasi* against Malaysia was not totally supported by internal groups particularly the Indonesian army (TNI-AD). This situation was used by the US to create the anti-Sukarno groups among the Indonesian Army. The intelligence operations under the CIA of the US, the Military Intelligence-Section 5 (MI5) of Britain, and Indonesian army intelligence were behind the 1965 coup and the collapse of PKI. Thirdly, the provocation of mass media towards Indonesia through the BBC, Radio Malaysia, Radio Australia and the VOA also played a key role (Easter 2001; & Roosa 2008:250–290).

It became clear that the Cold War situation leading to the rivalry between Soviet Union-US was a key factor in the 1965 coup. Furthermore, the real involvement of external powers through an intelligence operation had complicated the situation in Indonesian domestic politics particularly before the 1965 coup. It demonstrated that international structure in the Cold War was a determinant of the occurrence of the 1965 coup that brought an end to PKI existence in Indonesia.

### The 1965 coup and the reconciliation of relationship

Although the 1965 coup in Indonesia was a tragedy for Indonesia, it benefited Malaysia because it paved the way for reconciliation and normalize the relationship. The fall of Sukarno and succeeded by Suharto along with his military regime were a guarantee to the normalization of relationship because the Suharto administration was totally under capitalist bloc. Not surprisingly after the 1965 coup, two parties have similar characteristics particularly at the leadership level and the domestic politics after the collapse of PKI in Indonesia. Before *konfrontasi* Suharto has demonstrated a commitment to build the harmony of Indonesia-Malaysia relations. The arrival of the peace mission to Kuala Lumpur was the evidence that Suharto seriously wanted to end the *konfrontasi* with Malaysia.

It was interesting to note that the 1965 coup had produced an implication towards the complexity of domestic politics in the two countries. The complexity politics between the United Malays National Organization (UMNO) and the opposition groups, particularly with Pan-Malaysia Islamic Party (*Parti Islam Se-Malaysia*, PAS) were significant. During *konfrontasi* Dr. Burhanuddin of PAS took opposite position by supporting Indonesia. Dr. Burhanuddin opposed the establishment of Malaysia and supported Azahari’s rebellion in Brunei. Under Dr. Burhanuddin, PAS was closer to PKI in Indonesia and also supported the Malayan communist party (*Parti Komunis Malaya*, PKM) in order to challenge UMNO (Adam 1996). Two political factions also emerged in Indonesia during *konfrontasi* namely, pro-*konfrontasi* and anti-*konfrontasi*. Pro-*konfrontasi* encompasses Sukarno, nationalists, and PKI. While the anti-*konfrontasi* groups such as Indonesian Army (TNI-AD) and Islamic groups (Masjumi Party, Nahdlatul Ulama [NU] Party and sympathizers). During *konfrontasi* TNI-AD established a contact with Malaysian government and Indonesia citizens in Malay Peninsula and Singapore.

The 1965 coup and the rise of Suharto as Indonesian President significantly influenced the political changes in the both countries. In securing his power, Suharto and his military regime under the US support created a strategy to established GOLKAR party. Afterwards, the trans-party relations between GOLKAR of Indonesia and UMNO of Malaysia became the main instrument of Indonesia-Malaysia stable relations (UMNO-Online.com 2011)^e^. The 1965 coup not only paved the way for the Indonesia-Malaysia reconciliation, but also led to an end to *konfrontasi* and developed regional stability. The 1967 peace agreement held in Bangkok, Thailand, paved a new era of Indonesia-Malaysia relationship. Under this agreement both countries agreed to begin various cooperation such as social, economy, politics, and security.

The most significant achievement of the Indonesia-Malaysia relationship after the 1965 coup was the mass migration of Indonesian people to Malaysia in order to “support” Malayan political existences (Liow 2003). Since that time, the relations between both countries was harmonious. Many observers argued that under the Tun Razak leadership both countries enjoyed “special relationship (Hara 2008).”

### *Reformasi* and the fall of Suharto

#### The rise of *reformasi*

At the end of Suharto era, he was facing a similar situation compared to the Sukarno era. The occurrence of the Asian financial crisis in the late 1990s was utilized by Indonesian people to urge national political transformation. The Suharto regime was viewed as authoritarian and undemocratic and should be replaced by a new democratic government. In addition, the rise of globalisation after the end of the Cold War has strongly influenced this scenario. Suharto’s regime urged to implement a clean government, eradicate corruption, collusion, and nepotism. The three political issues namely corruption, collusion, and nepotism became a popular slogan at mass demonstrations and posed a real challenge to the Suharto administration. This phenomenon is considered quite acceptable and it has proven what Samuel Huntington had envisaged about “third wave of democratization” (Cabarello-Anthony 2005) after the end of the Cold War.

The international situation in the post Cold War era changed where the capitalist-communist issue became an obsolete matter and was replaced by globalisation issues. Globalisation brought a new issue which demanded states to become more transparent and accountable. Globalisation itself became popular after the end of the Cold War and demanded states to be more liberal in politics and economy. In terms of politics, globalisation urged states to establish a democratic and stable government. While in economic sector globalisation demanded states to be more liberal and go for open market policies. According to Waltz after the collapse of Soviet Union, globalisation becomes a major issue that indirectly promoted American values. Waltz argued that globalisation was constructed as “made in America (Waltz 1999).” It was because the US uses its political, economic and military leverage to manipulate international events to promote its interests (Waltz 1999:51).

The emergence of *reformasi* began with the occurrence of the Asian financial crisis in 1997 that brought to the rise of prices of basic needs. Demonstrations and social movements emerged. The efforts of government to overcome the crisis were no longer successful. The tensions increased between demonstrators and the security forces because the political responses from Suharto gave slowly. The situation worsened after Indonesian security forces took offensive action to deal with the demonstrators and the level of violence inevitably increased.

Jakarta as the centre of *reformasi* movement became a violent place and there were a lot of looting and damaging of public facilities. It reached a peak in May 12th, 1998 when Suharto decided to step down and was succeeded by his Vice President, BJ Habibie. Previously, in the campus of Universitas Trisakti in Jakarta, the security forces had attacked demonstrators and some of them were killed namely, Elang Mulya, Hafidin Royan, Hendriawan Sie, Hery Hartanto. While the riots in Yogyakarta, Moses Gatotkaca, a worker died in May 8, 1998 (Semanggi Peduli 2001-2003a)^c^. Some people have been lost, others arrested or murdered, because Suharto continuously to use the military action to deal with demonstration. Approximately, fifteen demonstrators have been lost during 1997 to 2001 and until recently their existence were still questionable (please see Semanggi Peduli 2001-2003b).

At the government level, BJ Habibie as a successor of Suharto has to settle for some tasks such as ending the *dwi fungsi* ABRI (Angkatan Bersenjata Republik Indonesia, Indonesian Armed Forces) (dual function of ABRI). The *dwi fungsi* means that the role of Indonesian Armed Forces not only in the security matter but also in the political and business arena. The *dwi fungsi* is the Suharto's strategy to securing his power in the parliament through the TNI/POLRI (Kepolisian Republik Indonesia. Indonesian Police Forces) committee (Fraksi TNI/POLRI). BJ Habibie urged to organise a democratic election immediately in order to establish a new democratic government. In addition, he has to arrange some important policies like reformation of mass media and urged to release some political prisoners in the Suharto era where many among them have been jailed since the 1965 coup (Habibie 2006). Actually, the democratisation issue became a challenge to Suharto because the *reformasi* itself was supported by the US. To the US, Indonesia has to reform and to be a more democratic country so to match the regional situations (Hadiz 2004).

#### Globalisation as cause of *reformasi*

The *reformasi* was the event determined by several factors that emerged in 1998. Firstly, the end of the Cold War with bipolar system followed by unipolar world and the emerging of new issues namely globalisation. The issues such as democracy, market liberalisations, and clean governance directly affected Indonesian domestic politics. Secondly, the pressure from the anti-Suharto groups during *reformasi* movement had demonstrated that international situation after the end of the Cold War was a significant factor in creating difficult situations in Indonesian domestic politics.

Overall, Suharto had to step down because he failed to keep up with the changes in international level notably globalisation and its consequences. The collapse of Suharto was the real evidence that the 1997 financial crisis is an international conspiracy which needed different responses and actions. Furthermore, the difficult political situations in Indonesia accompanied by financial crisis are really influences on Indonesia’s relations with Malaysia. This is because the Indonesian *reformasi* significantly influenced the Malaysian domestic politics which seriously challenged Mahathir's administration. This will be discussed in the next section.

#### The *reformasi* and the tension of relationship

The wave of *reformasi* followed by the resignation of Suharto in Indonesia was not done a single factor in the political transformation in Southeast Asia. Asian financial crisis and the “silent problems” among ASEAN countries became significant factors in the mapping of the political configuration in the region. However, the fall of Suharto followed by *reformasi* in the various sectors like liberalisation of mass media had significant affected the Indonesia's foreign relations particularly with Malaysia.

The Anwar Ibrahim case became a source of the tension in Indonesia-Malaysia relations because of the “interference” of Indonesian leaders in Malaysian domestic affairs. The *reformasi* in Indonesia had been attributed to Anwar to create a similar situation in Malaysia. However, he met the failure after was arrested by Malaysian police under Mahathir's authority. The fall of Suharto in Indonesia made Anwar more optimistic that he could make a similar *reformasi* agenda in Malaysia. Anwar was campaigning to fight against corruption, cronyism, and nepotism in the UMNO General Assembly in 1998. At the congress, Anwar planned to create no-confidence votes among UMNO members against Dr Mahathir (Sudin & Hussein 2002). The tension between Anwar-Mahathir was triggered by Anwar's provocative statement. In June 1998 Anwar said that “if we are unwilling to accept [political reform], we may face the Indonesian situation where people demanded changes” (Felker 1999).

The tension became more complicated when ASEAN leaders began to criticise Mahathir’s policy regarding the arrest of Anwar Ibrahim. ASEAN leaders recognised that the Anwar’s arrest is considered a human right abuse. BJ Habibie as a new Indonesian leader and the Philippine President Josef Estrada admitted sympathy to Anwar. Both leaders agreed to cancel in participation in the APEC general meeting to be held in Kuala Lumpur. Abdullah Ahmad Badawi as the Minister of Foreign Affairs gave a response promptly by saying that “if they will cancel, it will not be decreasing the ASEAN voices in the forum (Gatra 1998).” Afterwards, Abdurrahman Wahid (Gus Dur) as the fourth Indonesian President had admitted a sympathy to his colleague, Anwar Ibrahim. In his letter to Anwar, Gus Dur stated that, “greetings from Indonesia, and remember that we are behind your struggle. Remember that Allah is always with us (Gatra 1999).”

Through the Foreign Minister, Syed Hamid Albar the Malaysian government strongly opposed Gus Dur’s statement. Besides that, the Malaysian government also condemned Gus Dur because of his controversial statement published in the Far Eastern Economic Review (FEER). In December 9, 1999, FEER reported that, “Mahathir asked Gus Dur to assist him to approach with Israel.” However, Indonesian Foreign Minister Alwi Shihab said that Gus Dur never gave such a statement (Gatra 1999).

The Malaysian government worried about Gus Dur’s statement because this issue will impact to the UMNO’s voters in Malaysian general election in 1999. Former Indonesian Foreign Minister, Ali Alatas emphasised that Indonesia has to be careful in responding to the Malaysian domestic affairs. He described that the response of Indonesia and the Philippine leaders towards Anwar Ibrahim case as a personal sympathy and totally did not reflects the foreign policy of both countries (Gatra 1998).

In general, the above discussion showed that *reformasi* was influenced by the changes of Indonesia domestic politics which affected the foreign relations with Malaysia. Although the statements of BJ Habibie and Gus Dur were based on a personal sympathy, however they demonstrated that the bilateral relations had been disturbed. Specifically former Malaysian Foreign Minister, Syed Hamid Albar reminded that since Suharto as the Indonesian President, “the Indonesia-Malaysia relations were in harmony (ANTARA 2008).” The changes in Indonesian domestic affairs were also affected by the regional politics. The rivalry among ASEAN leaders was a sensitive issue and it contradicted the ASEAN situation during the Cold War particularly under Suharto in Indonesia.

## The role of systemic

To Indonesians, the 1965 coup and the *reformasi* were two events in different time, different situation, yet producing a similar result, namely the fall of autocratic government. Both events gave an implication to the regional politics where the 1965 coup paved the way for the Southeast Asian capitalist countries to cooperate with one another. The *reformasi* and the fall of Suharto led to the vacuum of leader in ASEAN after three decades under Indonesia and Suharto's dominant roles. It was undeniable that the demands of international situation were significant factor in driving of those events.

In the context of Indonesia-Malaysia relations both events were put on the table at a crucial moment. These events have interconnections and relatively occurred in the similar time and situation. The 1965 coup for instance, had close correlations with Malaysia and it really emerged because of the major power’s grand strategy. The Cold War situation was a determinant factor where Indonesia and Malaysia gained a strong influence from the major powers. Indonesia's close relations with communist countries totally contradicted with the Malaysia’s loyal policy towards the West. The British and the US as the main ally of Malaysia worried over Indonesia because of the Sukarno’s hard line policy and he was really un-cooperative.

Besides the US interest, the 1965 coup benefited Malaysia as a gateway to make peace approaches to Indonesia. However, without the US and British support, the 1965 coup could not have occurred. Both the US and British had maximised their resources to interfere in the Indonesian domestic politics, particularly to create an “internal ally” in Indonesia. It was interesting to note that Malaysian domestic politics divided into two blocs namely pro-West and anti-West. Pro-West groups namely UMNO and its sympathizers while anti-West/pro-Indonesia groups encompasses PAS and PKM.

The ideological disputes between capitalist and communist were the major factor of the occurrence of the 1965 coup. Indonesia was a major ally to Soviet Union and since the split of Sino-Soviet relations Indonesia built relations with China. Since independence, Malaysia has strongly been under the West influence notably British which are also pro-capitalist and anti-communist. The occurrence of the 1965 coup in Indonesia was the victory to the US and the ally. It was because their mission to save Indonesia from communist influence became successful. Not surprisingly ten days after the 1965 coup (coup occurred on October 1, 1965) New York Times wrote that, “U.S. is heartened by red setback in Indonesian coup (Roosa 2008:18–19).”

In the context of *reformasi* as a new event that occurred after the end of the Cold War a further analysis is possible. The occurrence of the Asian financial crisis in 1997 was an important factor in determining of the *reformasi*. However, the Asian financial crisis was the factor among many factors that merged together to urge Suharto to step down. The end of the Cold War followed by new situations (globalisation) era became a real challenge to Indonesian government. The new agendas such as democracy, liberalisation of market, transformation, and good governance had significant implications to Indonesia. Observers argued that globalisation was a new phenomenon after the end of the Cold War, however we agree with the Waltz’s argument that globalisation is arguably made by American (Waltz 1999). It was reality that the end of the Cold War with bipolar system followed by unipolar world remaining the US alone as the major power in international structure. The American superiority in the economics and overwhelming military power, tremendously assist in maintaining his survival in the unipolar world after the collapse of Soviet Union. At least, since the end of the Cold War until 2004, the U.S military expenditures were more than six time greater than those of prospective rivals such as Germany, Japan, France and Britain combined, so that no other state or even combination of states is capable to challenge and spread their influences, except with the U.S assistance (Ikenberry et al. 2009).

In the meantime, the situation in Malaysia was quite different compared with Indonesia where the Malaysian government (Mahathir) and all the stake holders were ready to face “attack” from the opposition (Anwar Ibrahim) and quite successful to survive in the crisis. Conversely, Suharto had to collapse because of the weakness of his infrastructure rather than his personality. Suharto finally had to move from his power after more than thirty two years ruling his military regime. It is probably different if the Cold War still exists because the major powers would play involved in both countries for security and strategic reasons. Because the Cold War ended and the international situation changed, Indonesia and Malaysia became less important to the major powers. In the Cold War era, however the major powers were noticeably involved in both countries because of the existence of Soviet Union along with the communist agendas. During the Cold War era wherever the Soviet Union and China were involved in some countries, the US and the allies were also ready to compete and fight against communism concurrently.

## Conclusion

The 1965 coup and the *reformasi*, the two moments that influence the Indonesia-Malaysia relationship was because these important events have interconnection with Malaysian domestic politics. After the 1965 coup both relationship dramatically changed because of the similar characteristic between Indonesia and Malaysia. Both countries have a similar agenda, vision and mission which were determined by the involvement of major powers in the Cold War arena. In contrast to this, *reformasi* that had affected Indonesia-Malaysia relations because of the lack in involvement of major powers and the international situation has changed. In addition, the similar characteristic between Suharto leadership style and his counterpart in Malaysian significantly influenced the harmonious relationship during the Presidency.

The split of domestic politics emerged in the both countries before the 1965 coup and the *reformasi* occurred. In the 1965 coup, Indonesian domestic politics had splits into two groups namely anti-Sukarno (TNI-AD and Islamic groups) versus pro-Sukarno (PKI and sympathisers). While in Malaysia domestic politics had splits into two political factions namely the anti-Malaysia (PAS and PKM) versus the government (UMNO and the British). In *reformasi*, Indonesian domestic politics had separate into two elements namely pro-new order (GOLKAR and TNI) challenged by anti-Suharto groups encompassing university students, scholars and the citizens. At the same time, the Malaysian domestic politics had split into two political groups, namely pro-government encompassing UMNO and the National Front (*Barisan Nasional*, BN) challenged by anti-Mahathir groups such as Anwar Ibrahim, PAS, and Democratic Action Party (DAP) under the Alternative Front (*Pakatan Rakyat*, PR).

These political configurations resulted in a new shape of the Indonesia-Malaysia relations. The 1965 coup produces a new leadership in Indonesia along with new paradigm which significantly influenced the close Indonesia’s relations with Malaysia. In the context of *reformasi* in 1998, the changes of Indonesian leadership influenced the Indonesian perspective towards Malaysia, though not significantly. Furthermore, the 1965 coup occurred in the bipolar era of the Cold War and on the contrary *reformasi* occurred in the unipolar world with the US as a single major power after the collapse of Soviet Union. Thus, the rise and fall of international structure situations significantly impact the international relations because Indonesia and Malaysia were the “smaller countries.” Automatically both countries will be affected by major power behaviours along with their high capability in international structure.

## Endnotes

^a^The four elements are executed as international structure and have to be measured as determinant factor to the states interest and behaviors. It adopted from Maksum 2011.

^b^TNI-AD stand for *Tentara Nasional Indonesia-Angkatan Darat* or Indonesian National Armed Forces-the Army

^c^It was also known as “*Tragedi Semanggi* [Semanggi Tragedy] and the victims also called as “*pahlawan reformasi* [reformation heroes]” (Please see Semanggi Peduli (2001-2003a) "Pahlawan Reformasi,” available in: http://www.semanggipeduli.com/Pahlawan/pahlawan.html (accessed 19 Jan 2010).

^d^See Roosa (2008:265), PRRI (*Pemerintahan Revolusioner Republik Indonesia* or Revolutionary Government of the Republic of Indonesia); Permesta (*Piagam Perjuangan Semesta* or Charter for Universal Struggle).

^e^At the present time, both Golkar and UMNO still built relationship even the Suharto had gone and Golkar not occupy Indonesian government. Some of UMNO leaders said that Golkar and UMNO has been work together since the early and continue to build a good cooperation (UMNO-Online.com 2011).
